# Urban Tree Pathogens: The Impact of *Neofusicoccum luteum* on *Araucaria heterophylla* (Norfolk Island Pine)

**DOI:** 10.1007/s00267-026-02633-8

**Published:** 2026-09-28

**Authors:** Anna Petrova, Chris Pratt, Ido Bar, Prabhakaran T Sambasivam, Ruby Naomi Michael

**Affiliations:** 1 Green Infrastructure Research Labs (GIRLS), Griffith Institute for Human and Environmental Resilience, Griffith University, Nathan, QLD Australia; 2 Present Address: School of Environment and Science, Griffith University, Nathan, QLD Australia; 3 School of Engineering and Built Environment, Griffith University, Nathan, QLD Australia; 4 Australian Rivers Institute, Griffith University, Nathan, QLD Australia

## Abstract

*Araucaria heterophylla* is a widely planted evergreen ornamental species in urban and coastal landscapes worldwide. Increasingly, these trees have been exhibiting stress symptoms, including canopy dieback, browning of foliage tips, defoliation, and reduced needle length. Previous research has shown that these symptoms may be the result of predisposing abiotic stressors, such as extreme weather events, soil compaction, and proximity to urban infrastructure. Building on this, the present study examines the trees’ foliar fungal communities and potential pathogens. Thirty-two (32) mature *A. heterophylla* trees of varying health growing on the foreshores of Gold Coast, Australia, were selected for isolation of fungi from foliage samples. Sixteen fungal species from ten families were identified. *Neofusicoccum luteum*, the most commonly represented species and previously unreported in *A. heterophylla*, was isolated from eight trees (25%) and selected for six-week-long pathogenicity testing under controlled glasshouse conditions. To assess pathogenicity, forty two-year-old trees were inoculated with either *N. luteum* or sterile agar (control). Half of these trees received limited water, and the other half was watered regularly. The infection rate exceeded 90%, and lesions developed in both groups, but lesions in the control groups were significantly smaller (*p* < 0.05) than in trees inoculated with *N. luteum*. Re-isolation at the end of the pathogenicity tests had a higher success rate in drought-affected trees (75%) despite the smaller size of the lesions. Although no mortality occurred, trees inoculated with the pathogen were significantly (*p* < 0.05) shorter than the control trees under the regular water conditions. These results suggest that *N. luteum* may contribute to *A. heterophylla* decline and reduced growth rate, but is unlikely to be the sole causal agent and is unable to kill a tree rapidly. Strategies for improving *A. heterophylla* health and management emerging from this study include the need for: (a) supplem.entary irrigation during prolonged drought to prevent the pathogen from entering through damaged bark; and (b) consistent tree health monitoring.

## Introduction

*Araucaria heterophylla* (Salisb.) Franco, commonly known as the Norfolk Island Pine tree, is an attractive evergreen species, typically featuring a single trunk and a distinctive pyramidal form (Gilman & Watson, 1993). It was introduced from Norfolk Island to mainland Australia in the late 18th century and subsequently became a widely planted species in coastal landscapes across several states, including New South Wales, Queensland, South Australia, and Western Australia. Many of these trees are now recognized for their heritage and cultural significance (National Trust of Australia, 2026). On the Gold Coast in Queensland, mature Norfolk Island pines that form a defining element of the coastal landscape are increasingly exhibiting symptoms of decline. The causes of this decline are currently poorly understood.

A number of physiological traits enable Norfolk Island Pine trees to thrive in coastal conditions, and it is widely planted outside of its home range on Norfolk Island. These traits include isohydricity - the ability to maintain a daily minimum leaf water potential constant regardless of the soil water potential (Zimmer et al., 2016) and halophytic properties – the ability to survive in environments with high salt concentrations in the rhizosphere (Zhang et al., 2010). Despite these traits, signs of *A. heterophylla* decline reported in coastal areas are dieback from the top, browning of foliage tips, general defoliation, and reduced length of branchlets (Dowden & Lambert, 1979; Moodie et al., 1986). Abiotic factors reportedly linked to this decline include elevated sodium levels due to surfactant exposure (Dowden & Lambert, 1979; Moodie et al., 1986), soil conditions, proximity to urban infrastructure, and extreme weather events (Petrova et al., 2025). Biotic factors (i.e. pathogenic infections) linked to decline include species from the genera *Lasiodiplodia* and *Neofusicoccum* (Darge, 2017; Golzar & Burgess, 2011; Huang & Wang, 2011).

Internationally, a substantial amount of information is available regarding the biotic threats posed to the health of *A. heterophylla*. Balocchi et al. (2022) published a comprehensive review of pathogens of Araucariaceae, which included this species. It was noted that pathogen activity can vary in severity: while some can cause major damage, others will not severely affect the health of the trees. Fungi from the Botryosphaeriaceae family were the most common pathogens identified in the review, and included *Botryodiplodia theobromae* (Raabe et al., 1981), *Diplodia seriata* (Tan et al., 2019), *Lasiodiplodia theobromae*, *Neofusicoccum mangiferae* (Huang & Wang, 2011), *Botryosphaeria parva* (Slippers et al., 2005), and *Neofusicoccum parvum* (Golzar & Burgess, 2011). Other pathogens found in foliage were *Phyllosticta drummondii, Phyllosticta acicola* (Kobayashi et al., 2003), *Phyllosticta concentric*a (Bissett & Palm, 1989), and *Mycoenterolobium platysporum* (Goos, 1970; Raabe et al., 1981).

Botryosphaeriaceae is a highly diverse family of ascomycetes with its species playing a variety of ecological roles. These fungi can act as endophytes, saprophytes, or pathogens, and are often found on woody plants. They are generally regarded as secondary opportunistic pathogens (Manawasinghe, 2016) and can remain latent for extended periods, becoming active only under conditions such as reduced precipitation and elevated temperatures (Batista et al., 2021; Phillips et al., 2013). Many Botryosphaeriaceae species known to cause problems in forestry and agriculture are latent, yet some of them are ubiquitous and are among the most aggressive endophytic pathogens (Slippers & Wingfield, 2007).

Abiotic factors such as prolonged hot and dry conditions, soil compaction, and physical damage can make trees more susceptible to infection by some members of the Botryosphaeriaceae (Bustamante et al., 2025). There is evidence that further changes in climate patterns may affect plant communities, making them more prone to damage (Slippers & Wingfield, 2007). In cities, weather extremes, urban infrastructure, air quality, and soil conditions may create additional stresses (Locosselli et al., 2019; Petrova et al., 2025).

This study investigated the fungal communities associated with declining *A. heterophylla* and identified species that have the potential to cause significant damage to these trees. The research aimed to inform management strategies for declining Norfolk Island pines on the Gold Coast and provide insights relevant to other coastal regions worldwide where the species is valued as a heritage or character tree. Specifically, the study aimed to (1) isolate fungal species associated with declining mature *A. heterophylla* trees and (2) evaluate the pathogenic potential of the most prevalent species, *Neofusicoccum luteum*.

## Materials and Methods

### Study Context

This study was conducted on the Gold Coast, South East Queensland, Australia, a region that experiences a warmer and wetter climate than the native habitat of *Araucaria heterophylla* on Norfolk Island. Mean annual rainfall at Coolangatta (weather station No. 040717) is 1517.4 mm, compared with 1282.7 mm on Norfolk Island (weather station No. 200288). Average monthly temperatures on the Gold Coast range from 20.7 °C in July to 28.5 °C in January, exceeding the milder 18.4 °C to 25.0 °C range observed on Norfolk Island (BoM, 2025). Temperature extremes also differ markedly: summer maximums on the Gold Coast can reach 40 °C, whereas the highest recorded temperature on Norfolk Island is 28.5 °C (BoM, 2025).

In the years preceding this study, particularly prior to 2023, the Gold Coast experienced periods of both reduced rainfall and elevated temperatures. Notably, 2019 was among the driest years on record, receiving only 825.6 mm of rain - just above the 1986 record low of 792.6 mm. Rainfall in 2018 was also below average at 1046.2 mm, and another markedly dry year in the region’s recent history was 2013, with 994.2 mm recorded.

Temperature anomalies accompanied these dry conditions. In both 2019 and 2017, peak daily summer temperatures exceeded typical monthly averages. Although the long-term mean maximums are 28.5 °C for January and 27.5 °C for December, temperatures in 2019 rose to 30.1 °C and 29.2 °C, respectively. Annual mean temperatures were similarly elevated, reaching 25.9 °C in both 2017 and 2019 (BoM, 2025).

During this period, many of the Gold Coast’s Araucaria trees were “crown lifted” – their lower branches were pruned – during the 2018 Commonwealth Games for better visibility (M. Drury, personal communication, August, 19, 2025).

### Tree Sampling and Pathogen Isolation

Foliage of 32 *A. heterophylla* trees, growing along the Gold Coast, Australia, and exhibiting varying levels of decline, was sampled for fungal isolation. All samples had visible signs of browning and were collected from the lower branches, accessible from the ground, with a few exceptions where an aerial work platform was required due to extensive loss of lower foliage. Ninety-six fragments, comprising three approximately 1 cm-long branchlet segments with leaves from each tree were surface-sterilized for 2 min with 1.5% Sodium hypochlorite, triple rinsed in sterile water, placed on Potato Dextrose Agar (PDA) in 9 cm Petri dishes, and incubated at 25°C for 2-3 weeks (Aguilar-Pérez et al., 2023; Arnold et al., 2000). The plates were assessed every three days; isolates were sub-cultured, transferred onto new media, and grown for an additional two weeks.

### DNA Extraction

High molecular weight DNA was extracted from fungal isolates using the modified Cetyltrimethylammonium bromide (CTAB) DNA extraction protocol (Carr, 2022) for plants and fungi.

Briefly, 50–60 mg of two-week-old mycelium samples were collected and ground under liquid nitrogen with sterile mortar and pestle. Ground samples were mixed with 800 µL of CTAB buffer in 2 mL tubes. 60 µL of 10% N-LNS, 60 µL of 10% PVP, and 40 µL of 10% SDS were mixed with the sample and incubated at 65°C for 30 min. 800 µL of KCl were added to the samples and centrifuged at 13000 rpm for 15 min. 600 µL of upper aqueous solution was then transferred into a 1.5 mL tube, mixed with 750 µL of PCI, and centrifuged for 15 min. 600 µL of upper aqueous was then mixed with 80 µL of 3 M Na-acetate, and 800 µL of cold Isopropanol for DNA precipitation.

The samples were incubated on ice for 30 min and centrifuged for 5 min at 13000 rpm. Once the upper aqueous solution was discarded, DNA pellets were washed twice with 800 µl of 70% ethanol for about 1 min to remove PCI traces, and then centrifuged for 15 min at 13000 rpm. The ethanol wash solution was then discarded, and samples were dried at room temperature for 30–60 min. The DNA pellet was then eluted in 50 µl of sterile MiliQ water and incubated at 45°C for 1 h. 5 µl RNAse was added for RNA digestion, and samples were incubated at 37°C for 15 min. DNA integrity, purity and quantity were assessed by gel electrophoresis, Nanodrop and Qubit, respectively.

### DNA Sequencing and Bioinformatics Analysis

Nanopore sequencing was selected for initial fungal identification as it offered the best chance to quickly classify the pathogen at a genus level, and to explore whether it could potentially be applied for field-diagnostics (Chalupowicz et al., 2019). The DNA was prepared for long-read Nanopore sequencing following the standard protocol by removal of contaminants (Qiagen DNeasy PowerClean CleanUp Kit) and short fragments (ONT Short Fragment Eliminator Kit, EXP-SFE001), followed by ligation of sequencing adapters using the Rapid Barcoding Kit 24 V14 (ONT SQK-RBK114.24). The libraries were then sequenced on an Oxford Nanopore Mk1C with a MINFLO114 flowcell (R10).

Bioinformatics processing and analysis of the sequencing reads was performed on the Griffith University Gowonda HPC Cluster. Sequencing data was first demultiplexed with Guppy and the resulting fast5 files were converted to pod5 file format (using pod5 tools v0.2.3, see the documentation) and then basecalled with Dorado v0.5.3+d9af343 with the ‘dna_r10.4.1_e8.2_400bps_sup@v4.2.0’ model.

The basecalled reads were then taxonomically classified with Kraken2 v2.1.3, using the entire NCBI ‘nt’ database as a reference and with the ‘--confidence 0.51 --minimum-base-quality 20’ parameters (Lu et al., 2022). The classification for each barcode (sample) was summarized, showing the taxon with the highest score and the one with the highest number of matching read fragments (hits). Reads with matches of interest were then matched back against the NCBI ‘nt’ database using reciprocal BLASTn v2.16.0 (Camacho et al., 2009) to confirm the taxonomic classification.

Samples with potentially pathogenic genera or those with poor DNA quality were submitted to Grow Help Australia - a plant pest and disease diagnostic service operated by the Queensland Government’s Department of Primary Industries - to confirm species. For identification, mycelium was scraped from the surface of the cultures, and total genomic DNA was extracted using the BioSprint® DNA Plant Kit (Qiagen) according to the manufacturer’s instructions. Each 30 μl reaction contained 0.3U Phusion® High-Fidelity DNA Polymerase (New England Biolabs), 1 × Phusion HF Buffer, 0.2 mM dNTPs, and 0.5 μM of each primer.

PCR cycling conditions were as follows: initial denaturation at 98°C for 30 s, then 30 cycles of 98°C for 15 s, Tm°C for 15 second and 72 °C for 30 s, and a final 5 min extension at 72°C (Table 1). Unpurified PCR products were submitted to the Australian Genome Research Facility (AGRF) for dual-direction Sanger sequencing. Full sequence reads were trimmed and analyzed using Genieous Prime Software v2019.0.4 (Biomatters Ltd.) Sequences were compared to the GenBank database using BLASTn.

**Table 1:** Primers used for PCR amplification and sequencing.

Marker	Primer	Sequence (5′–3′)	Tm (°C)	Reference
ITS	UN-UP18S42	CGTAACAAGGTTTCCGTAGGTGAAC	62	Schroeder et al. (2013)
	UN-LO28S576B	CTCCTTGGTCCGTGTTTCAAGACG		Schroeder et al. (2013)
tef	EF1-728	CATCGAGAAGTTCGAGAAGG	59	Carbone & Kohn (1999)
	EF2R	GGARGTACCAGTSATCATGTT		O’Donnell et al. (1998)
β-Tubulin^a^	T1-F	AACATGCGTGAGATTGTAAGT	62	O’Donnell & Cigelnik (1997)
	Bt2c-R	CTCAGTGTAGTGACCCTTGGC		Glass & Donaldson (1995)
	T2-R	TAGTGACCCTTGGCCCAGTTG		O’Donnell & Cigelnik (1997)
gpd	gpd1	CAACGGCTTCGGTCGCATTG	65	Berbee et al. (1999)
	gpd2	GCCAAGCAGTTGGTTGTGC		Berbee et al. (1999)

^a^Initial PCR completed with T1/Bt2cR, seq reaction completed with T1/T2

### Pathogenicity Test

To establish the causal relationship between the suspected pathogen and tree decline symptoms, Koch’s postulates were applied (Fredericks & Relman, 1996; Koch, 1890). For that, forty healthy two-year-old *A. heterophylla* trees were selected for a pathogenicity test from the City of Gold Coast nursery, where they were growing prior to the experiment. The trees were assessed, measured, and placed in a glasshouse equipped with two evaporative coolers at the EcoSciences Precinct, Brisbane, for 6 weeks. The trees were sourced from a local nursery where they were growing in a potting mix consisting of 80% composted bark, 17% coco peat, and 3% gypsum, with the addition of slow-release fertilizer. They were separated into four groups: (1) Inoculated with *N. luteum* and watered regularly, (2) Inoculated with *N. luteum* and exposed to drought, (3) Control, inoculated with sterile agar and watered regularly, (4) Control, inoculated with sterile agar and exposed to drought.

An *in-planta* stem inoculation assay was performed in the glasshouse on two-year-old potted *Araucaria heterophylla* trees growing in 250mmØ pots. Each tree was visually divided into three parts along the stem, and each part was inoculated with an isolate of *N. luteum* of the Botryosphaeriaceae family (NCBI accession number PRJNA1492497). Inoculation of each part was done by making a shallow 2 cm long wound with a scalpel. A mycelial plug, approximately 3 mm by 3 mm, was inserted under the bark, covered with a sterile wet cotton square, and wrapped in parafilm to prevent desiccation.

The point of inoculation on each branch was surface sterilized by spraying with a 70% ethanol solution, allowing to air-dry before using the wound inoculation method with colonized agar plugs, as described above. Plants inoculated with uncolonized PDA plugs served as control. The length of the necrotic lesions under the bark was observed and measured at the end of six weeks post-inoculation.

After inoculation, all trees were watered every second day with 700 ml of water for 10 days, then watering was permanently discontinued for Groups 2 and 4, while Groups 1 and 3 continued to receive water until the end of the experiment. The temperature in the glasshouse was recorded daily with KIMO STH-ID 150 temperature probe located in the center of the room and calibrated by the manufacturer’s technician every 6 months.

The height of the trees was measured from base of the plant to the growing tip at the beginning and at the end of the experiment. Data was collected at the end of the 6-week long experiment, and the length and width of all the lesions were measured with a ruler and analyzed. Where lesions were larger than the original size of the cut, more bark was removed to be able to measure them.

### Re-isolation and Confirmation of *N. luteum*

In order to fulfill Koch’s postulates, isolations from resulting necrotic tissues were cultured on PDA plates, and identity of the fungal species was confirmed morphologically and by DNA sequencing, as detailed below.

Four trees from each group were randomly selected for re-isolation to verify that the lesions were caused by *N. luteum*. Small parts of the trunk containing lesions were sterilized for 2 min with 1.5% Sodium hypochlorite, triple rinsed in sterile water and placed on PDA plates. Fungi with characteristics of *N. luteum* were sub-cultured, grown for one week, and their DNA was extracted following the methodology described above. The extracted DNA was sent to AGRF for dual-direction Sanger sequencing. As previously, full sequence reads were trimmed, analyzed, and compared to the NCBI GenBank non-redundant nucleotide database (nt) using BLASTn (Camacho et al., 2009).

### Statistical Analysis

Differences in plant height and lesion size among treatments were analyzed in R v. 2023.09.0 (R Core Team, 2025). For plant height, treatment effects were initially evaluated using one-way analysis of variance (ANOVA) (Fisher, 1925). Model assumptions were assessed by examining residual normality using the Shapiro–Wilk test and quantile–quantile (Q–Q) plots (Shapiro & Wilk, 1965), and homogeneity of variances using Bartlett’s test (Bartlett, 1937). As the assumption of homogeneity of variances was violated, Welch’s one-way ANOVA was used to compare mean heights among treatments (Welch, 1951). To complement the parametric analysis, a Kruskal–Wallis test was also performed to assess differences among treatments without assuming normality (Kruskal & Wallis, 1952). Where significant differences were detected, pairwise comparisons were conducted using Wilcoxon rank-sum tests, with *p*-values adjusted using the Benjamini–Hochberg (BH) procedure to control for multiple comparisons (Benjamini & Hochberg, 1995).

### Data Visualization

Change in height and lesion size across treatment groups were visualized using box-and-whisker plots generated with the ggplot2 package (Wickham, 2016). Data points were shown using jittered points to improve visibility of individual measurements.

## Results

### Isolated Fungi

**Fig. 1:**
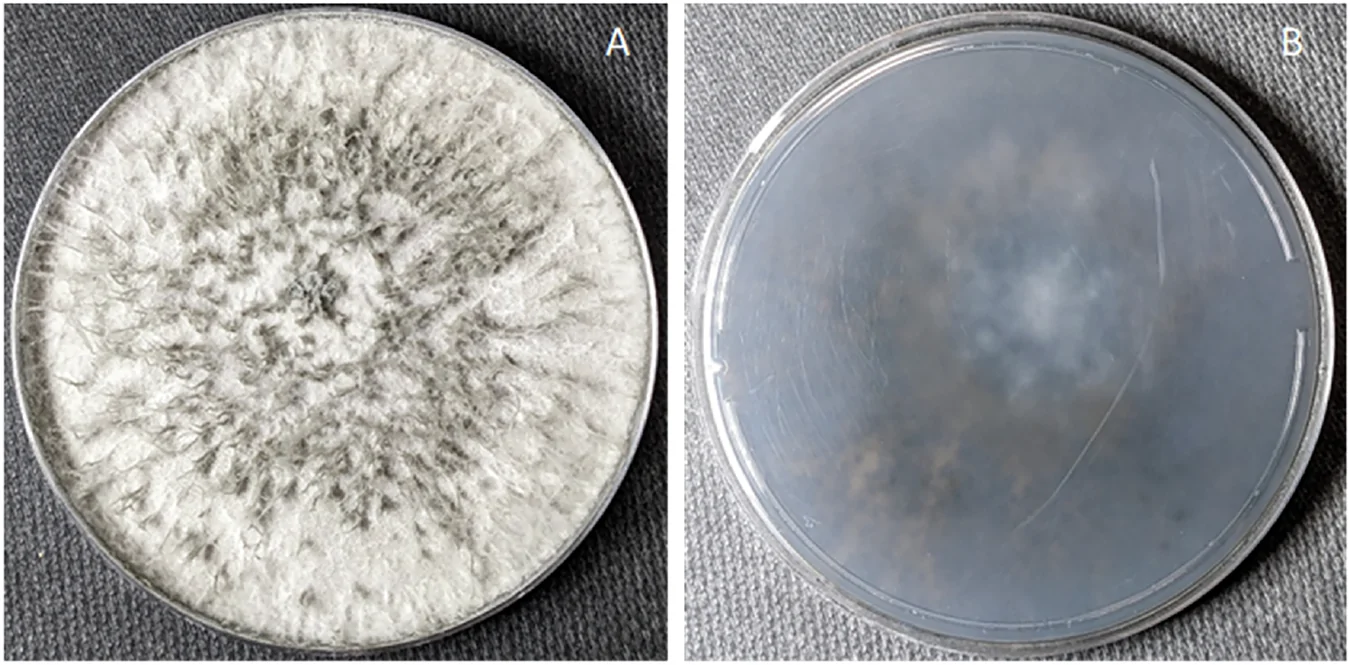
*Neofusicoccum luteum* isolate obtained from the symptomatic trees on Potato Dextrose Agar (PDA) medium after 14 days. **A** Top of the fungus/plate, **B** Bottom of the fungus/plate

Sixteen fungal species representing ten genera were isolated from foliage samples. Thirteen species were identified to the genus level using Nanopore sequencing. Sequencing of the remaining three species was unsuccessful; consequently, these isolates, together with two species of particular interest, were submitted to Grow Help for further identification. A literature review was undertaken to identify known pathogens associated with *A. heterophylla* among the isolated fungi. Three genera were found to include known pathogens, with isolates of *Neofusicoccum* and *Alternaria* further identified to the species level (Table 2). Of these, *N. luteum* (Fig. 1), isolated from eight trees, was selected for pathogenicity testing due to its prevalence and known pathogenic potential.

**Table 2:** Fungi found in the foliage of mature *A. heterophylla* and the number of trees they were isolated from (NCBI accession umber PRJNA1492497)

Genus	Number of trees	Confirmed species or the number of isolated species	Genes sequences completed by Grow Help
*Neofusicoccum*	8	*Neofusicoccum luteum*	ITS/tef/btub/RPB2
*Nigrospora*	8	One species	tef
*Pseudopithomyces*	6	Two species	
*Trichoderma*	6	Three species	ITS
*Pestalotiopsis*	5	One species	ITS
*Clonostachys*	4	Two species	ITS, ITS/tef
*Fusarium*	2	One species	
*Alternaria*	2	*Alternaria alternata* complex species and two unidentified species	Gpd, ITS
*Paraeutypella*	1		ITS/btub
*Aspergillus*	1	One species	

### Pathogenicity Tests

The experiment was conducted in a glasshouse in the middle of the Australian summer (December-January) when the temperatures are at their highest. The glasshouse had evaporative coolers, and the temperature during the six weeks of the pathogenicity test fluctuated between 18.6°C (10.30 pm) and 34.2°C (1.40 pm).

**Fig. 2:**
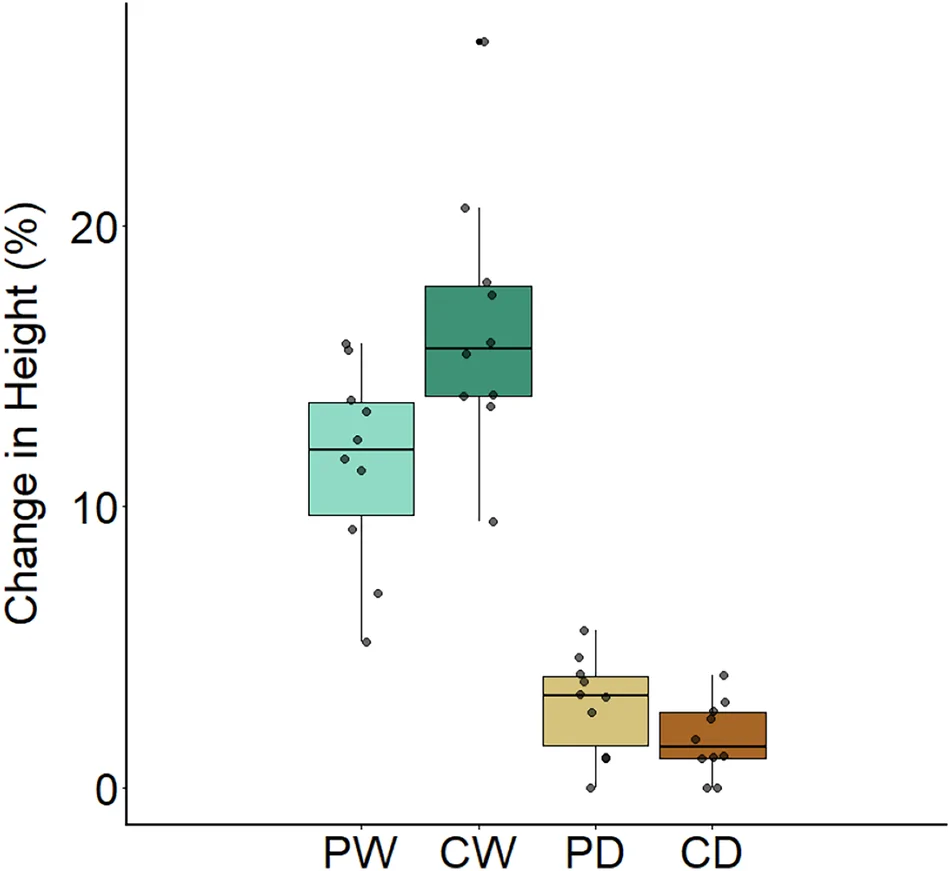
Difference in tree height at the beginning of the experiment and at the end, shown in percent change for four groups: trees inoculated with *Neofusicoccum luteum* and watered (PW), inoculated with *Neofusicoccum luteum* and subjected to drought (PD), inoculated with sterile agar and watered (CW), and inoculated with sterile agar and subjected to drought (CD)

By the end of the experiment, trees inoculated with sterile agar and maintained under well-watered conditions were the tallest, followed by *N. luteum*-inoculated well-watered trees, *N. luteum*-inoculated drought-stressed trees, and sterile agar-inoculated drought-stressed trees. Pairwise comparisons adjusted for multiple testing using the Benjamini-Hochberg procedure revealed significant differences in height among most treatment groups. Trees inoculated with *N. luteum* under watered conditions were significantly shorter than sterile agar-inoculated watered trees (*p* = 0.0107), but significantly taller than both *N. luteum*-inoculated, drought-stressed trees (*p* < 0.001) and sterile agar-inoculated, drought-stressed trees (*p* < 0.001). Similarly, sterile agar-inoculated well-watered trees were significantly taller than both drought-stressed groups (*p* < 0.001 and *p* < 0.001 respectively). In contrast, tree height did not differ significantly between *N. luteum*-inoculated drought-stressed trees and sterile agar-inoculated drought-stressed trees (*p* = 0.121) (Table 3, Fig. 2).

**Table 3:** Difference in tree height at the beginning of the experiment and at the end, shown in percent change for four groups: trees inoculated with *Neofusicoccum luteum* and watered, inoculated with *Neofusicoccum luteum* and subjected to drought, inoculated with sterile agar and watered, and inoculated with sterile agar and subjected to drought

	Pathogen-Watered, % change	Pathogen-Drought, % change	Control-Watered, % change	Control-Drought, % change
**Average**	11.51876	2.937272	16.48734	1.726103
**±SD**	3.513504	1.757187	4.643793	1.321461

**Fig. 3:**
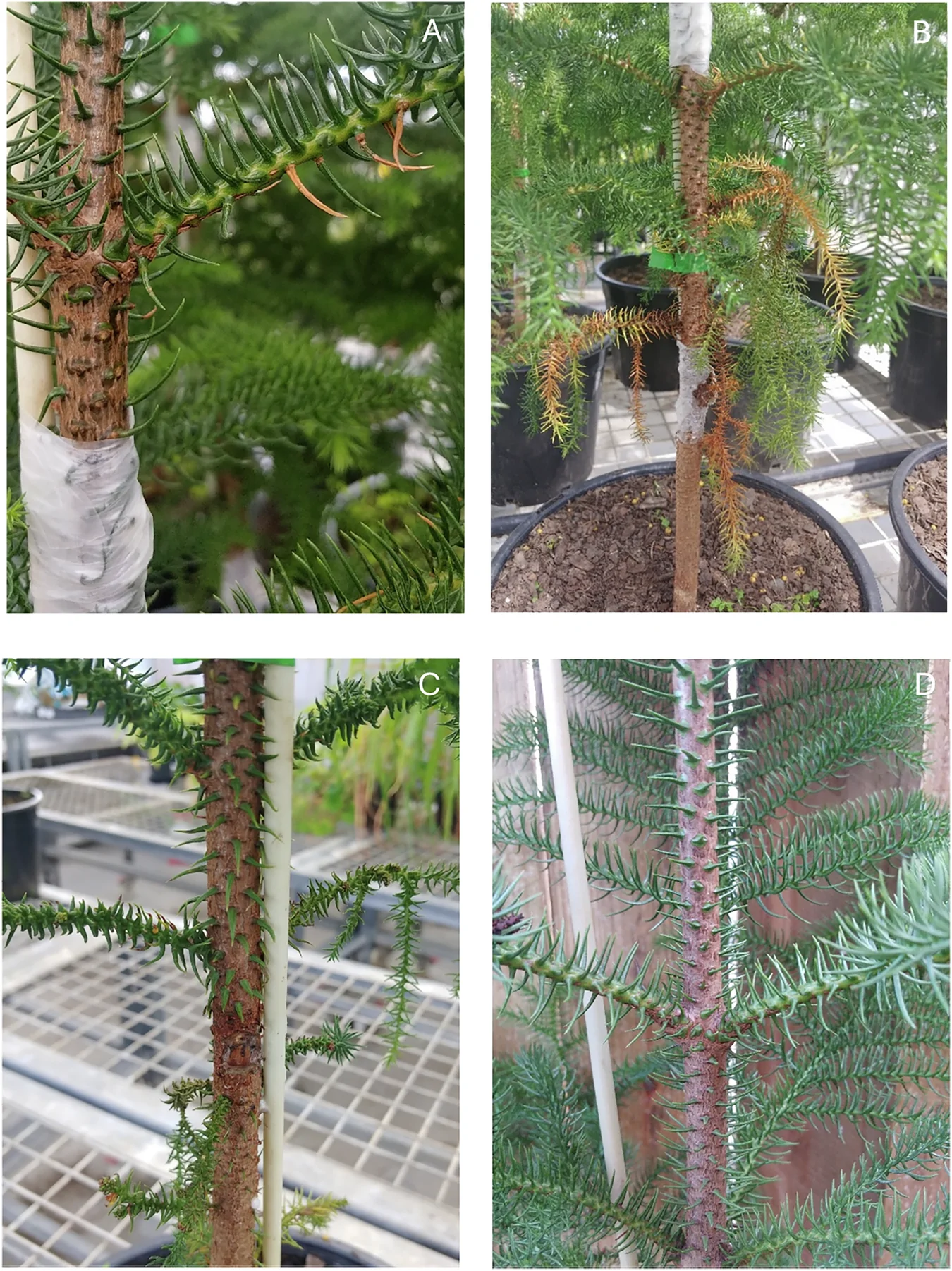
Visual change in *Araucaria heterophylla* trees inoculated with *Neofusicoccum luteum* agar at the end of the pathogenicity test. **A** Needle browning under regular watering regime, **B** Extensive needle and branch browning in trees in drought condition, **C** Leaves shrinking in trees under regular watering regime, **D** Healthy foliage of a tree prior to inoculation shown for comparison

All trees, including both pathogen-inoculated and control groups, exhibited minor needle browning near the wound sites (Fig. 3 A, B). In each group, two trees displayed more extensive browning, localized primarily at the lower sections of the trunk, while the upper foliage remained unaffected and visibly healthy. Additionally, one tree in each infected group exhibited progressive foliage shrinkage, extending from the base to the apex of the tree (Fig. 3C).

The infection rate in pathogen-inoculated trees was 90% under regular watering and 93% under drought conditions. *N. luteum* was re-isolated from 75% of trees subjected to drought and from 25% of regularly watered trees. Fungi other than *N. luteum* re-isolated from lesions were visually different from the target species and were not identified. Therefore, Koch’s postulates were only partly met.

**Fig. 4:**
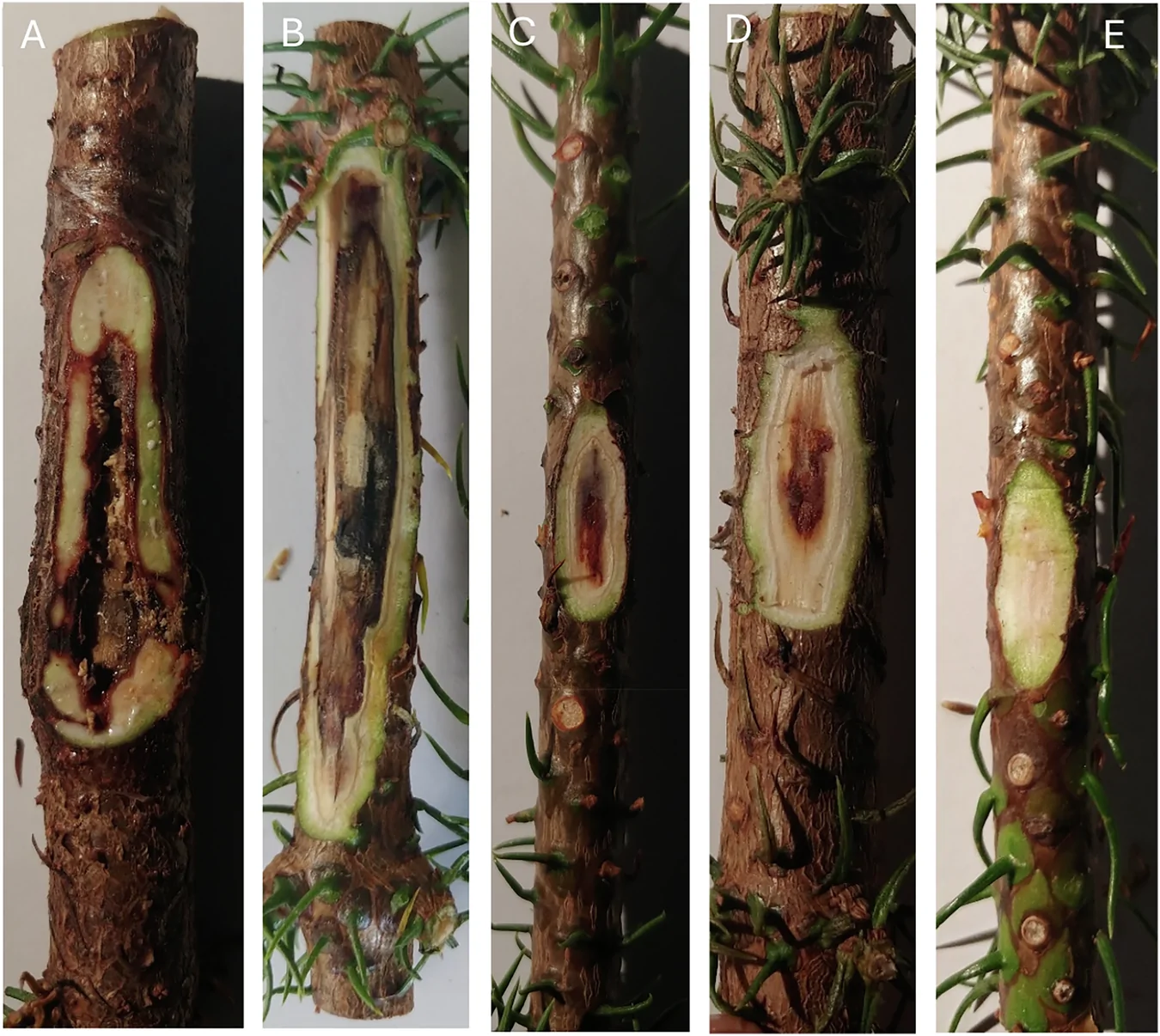
Examples of lesions on *Araucaria heterophylla*: trees inoculated with *Neofusicoccum luteum* and regularly watered **A**, trees inoculated with *Neofusicoccum luteum* and subjected to drought trees **B**, trees inoculated with sterile agar and regularly watered trees – control **C**, trees inoculated with sterile agar and subjected to drought trees **D**, tree inoculated with sterile agar without symptoms **E**. The cut was extended during the examination, where the lesions were larger, to be able to measure the size

**Fig. 5:**
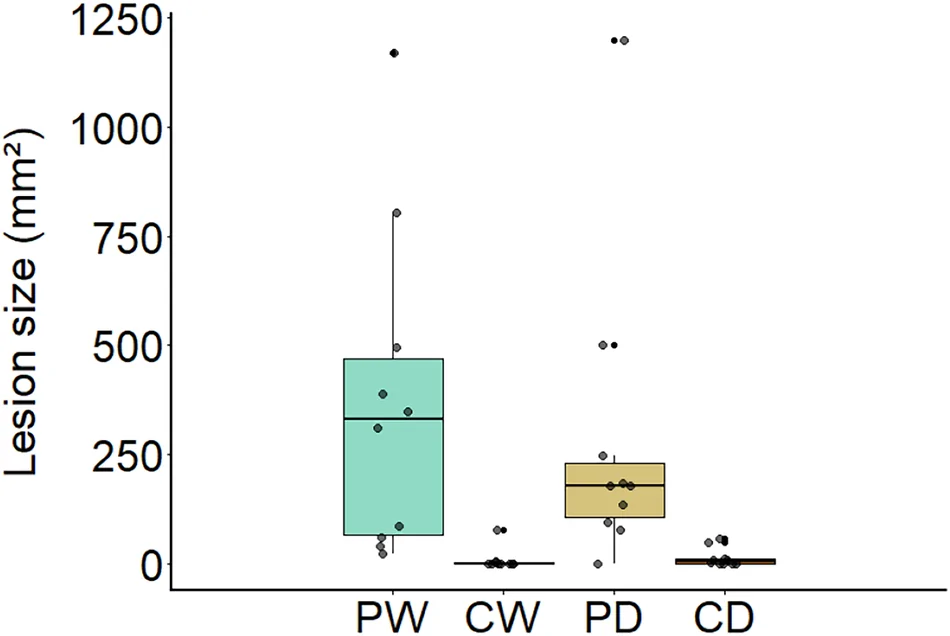
The size of the lesion areas (length x width) of *Araucaria heterophylla* trees six weeks post-inoculation. PW – inoculated with *Neofusicoccum luteum* and watered regularly (Pathogen Watered), CW – inoculated with sterile agar and watered regularly (Control Watered), PD - inoculated with *Neofusicoccum luteum* and subjected to drought (Pathogen Drought), CD – inoculated with sterile agar and subjected to drought (Control Drought)

Across all treatments, trees inoculated with *N. luteum* developed significantly larger stem lesions than trees inoculated with sterile agar (Table 3). Pairwise comparisons indicated that lesion size was significantly greater in both watered and drought-stressed *N. luteum*-inoculated trees than in sterile agar-inoculated controls (BH-adjusted *p* ≤ 0.003). In contrast, lesion size did not differ significantly between watered and drought-stressed trees within the *N. luteum*-inoculated treatment (BH-adjusted *p* = 0.739), indicating that drought stress did not significantly influence lesion development following inoculation (Tables 4, 5; Figs. 4, 5).

**Table 4:** Calculated average and standard deviation of the length and width of lesions developed after inoculations with *Neofusicoccum luteum* under regular watering regime and under water stress and after inoculation with sterile agar under regular watering regime and under water stress

	*N. luteum* + water (mm)		*N. luteum* + drought (mm)		Agar + water (mm)		Agar + drought (mm)	
	Length	Width	Length	Width	Length	Width	Length	Width
**Average**	15.97	4.62	16.70	4.59	1.23	0.35	2.25	0.72
**±SD**	13.56	4.36	16.42	2.50	3.27	0.88	3.95	1.16

**Table 5:** Stem lesions from different treatment groups, comparisons showing significant BH-adjusted *p*-values

Comparison	Significant BH-adjusted *p*-values
*N. luteum* + watered vs. sterile agar + watered	0.002
*N. luteum* + watered vs. sterile agar + drought-stressed	0.002
Sterile agar + watered vs. *N. luteum* + drought-stressed	0.003
*N. luteum* + drought-stressed vs. sterile agar + drought-stressed	0.003

## Discussion

Of the fungi isolated from *A. heterophylla*, three taxa were considered potentially pathogenic: *Nigrospora* species, *Neofusicoccum luteum*, and *Alternaria alternata*. *N. luteum* was not previously isolated from *A. heterophylla*, while *Nigrospora* species and *A. alternata* are not known to cause serious disease in these trees.

The *Nigrospora* isolate was not identified to species level, but the genus contains species capable of causing plant disease. *N. lacticolonia*, for example, was previously reported to cause brown spot disease in some trees, crops, and ornamental plants. It is not known to cause tree mortality, yet it can cause some damage. Among the reported plants were dragon fruit and bougainvillea, with symptoms expressed as regular or irregular reddish-brown spots (Kee et al., 2019; Li et al., 2022).

Another identified pathogen, *A. alternata*, has a wide host range (DeMers, 2022), causing leaf spot disease in a variety of plants, including fruit trees (Peever et al., 2002) and conifers (Zhang et al., 2023). Infected plants appear to have leaf blight, leaf spot, and black leaf tips (Zhang et al., 2023).

While the two pathogens mentioned above can affect tree aesthetics and reduce productivity, they are unlikely to cause tree mortality. Therefore, this study focused on the third fungal species, *N. luteum*, an opportunistic pathogen belonging to the Botryosphaeriaceae. Although members of the Botryosphaeriaceae typically infect stressed or weakened hosts, they can cause extensive stem cankers, branch dieback, and tree decline, and are among the most frequently reported secondary fungal pathogens in urban environments (Petrova et al., 2025a).

*N. luteum* was not previously documented in *A. heterophylla* (Balocchi et al., 2022), however, it was reported in a variety of other trees and shrubs. For example, it was found to affect *Rhododendron* spp. in Spain (Pintos Varela et al., 2011), tejocote (*Crataegus mexicana*) in the US (Adesemoye et al., 2013), oak trees (*Quercus robur*) in Portugal (Barradas et al., 2013), Pinot noir grapevines in New Zealand (Amponsah et al., 2014), and olive trees (*Olea europaea*) in Australia (Sergeeva et al., 2009). Some of the symptoms include fruit rot, leaf necrosis (Sergeeva et al., 2009), branch dieback, and canker (Fernandes et al., 2021).

To establish *N. luteum* as a causal agent of *A. heterophylla* decline, Koch’s postulates must be fulfilled (Brock, 1999). The pathogen (a) has to be present in every case of the disease, (b) can be isolated from the diseased host and grown in pure culture, (c) is capable of causing the disease when introduced into a new host, and (d) can be re-isolated from the new host.

*N. luteum* was isolated from eight mature trees out of thirty-two, and its re-isolation from the young inoculated trees produced mixed results. While inoculation resulted in lesions, the pathogen was isolated from 75% of trees that were affected by the drought treatment, and the number was lower for the watered group (25%). This may be due to the secondary invaders and opportunists not identified in this study that outcompeted *N. luteum*. Since Koch’s postulates were only partially fulfilled, the findings suggest that *N. luteum* contributed to the decline of *A. heterophylla*, although a causal relationship could not be conclusively demonstrated.

It is worth noting that the drought stress resulted in stunted tree growth for both infected and control groups, yet the inoculated trees were affected more and grew less on average, even when watered regularly. The lesions extended into xylem tissue and may have impaired the transport of water and nutrients (Zimmermann, 1982).

The relatively high infection rate of *N. luteum* in *A. heterophylla* observed in this study, together with its effects on lesion development and growth rate, highlights the need for preventative management strategies. Prolonged drought conditions, to which the trees were exposed on the Gold Coast prior to the study, may have stressed the host and promoted the transition of latent endophytic *N. luteum* infections to active disease, rather than solely facilitating infection (Cameron, 2019).

Although *A. heterophylla* exhibits isohydric behavior (Zimmer et al., 2016), its drought tolerance is limited. Supplementary irrigation may therefore be necessary, depending on factors such as tree size and soil type. In this experiment, two-year-old trees grown in a potting mix exhibited signs of wilting after three weeks without watering. The study was conducted during summer, when temperatures occasionally exceeded 30 °C and reached a maximum of 34.2 °C. These conditions may have contributed not only to increased infectivity but also to accelerated dehydration rates.

Successful tree water management can be achieved through proactive planning for prolonged drought events (Cremades et al., 2021). Where feasible, soil improvement should be considered to enhance water-holding capacity (Lin et al., 2018). Additionally, as crown lifting creates wounds and potential infection pathways, the necessity of this practice and its associated risks should be carefully reassessed.

Currently, no universal and straightforward treatment exists for *Neofusicoccum* species, though various control measures are under investigation, including fungicides and antagonistic microorganisms (Langa-Lomba et al., 2023; Pollard-Flamand et al., 2022; Ayres et al., 2022). Previously, several studies were conducted on a different woody plant - the grapevine - and explored potential biocontrol agents for disease management with varying degrees of success. *Bacillus velezensis* demonstrated a protective effect against *N. parvum* and *D. seriata* (Langa-Lomba et al., 2023), while *Trichoderma* species (*T. asperelloides, T. atroviride, and T. canadense*) provided protection against *D. seriata* and *N. parvum* for up to 21 days (Pollard-Flamand et al., 2022). Fungicidal treatments, including tebuconazole, pyraclostrobin, and fluazinam, were also tested, providing control for up to 14 days when applied within six days post-inoculation (Ayres et al., 2022). Currently, frequent application of fungicides and antagonistic microorganisms is impractical for mature *A. heterophylla* trees due to their height and their occurrence in public settings.

Once an infection spreads, its control becomes increasingly difficult to achieve. While treatment can be used on vines, tall trees like *A. heterophylla* require a different approach, and targeting prevention could give them a better chance of survival (Štůsková et al., 2023). Adequate water availability has previously been identified as an important factor in reducing susceptibility to infection (Jan et al., 2011), and while this research showed that lesions were larger in regularly watered trees, reintroduction of water after the drought exposure was not tested, which could potentially worsen the outcome.

It is expected that climate change will add stress on trees in forestry and agriculture through extreme weather events, which may, in turn, increase the abundance and potency of pests and pathogens (Slippers & Wingfield, 2007). Cities, with their additional pressures, may be affected even more. Therefore, the capacity of trees in urban areas to mitigate air pollution, create shade and encourage healthy lifestyles through adding comfort and reducing stress may be compromised (Turner-Skoff & Cavender, 2019). The protection of mature trees and the selection of those that are drought-tolerant and less susceptible to pathogens is crucial if cities are to remain green.

## Limitations

Fungal isolation was conducted on foliage collected from the lower branches of mature trees, which may have excluded fungal species present in the upper canopy due to the access constraints. Although three pathogens were isolated, pathogenicity testing focused solely on *N. luteum* because it was considered the most aggressive species and of greatest management concern. Consequently, the pathogenic potential of *Nigrospora* and *Alternaria* species remains unknown and should be investigated in future studies, both individually and in combination, to determine their role in the decline of *A. heterophylla*.

The pathogenicity experiment was conducted over six weeks in a glasshouse pot trial using young trees, with a subset subjected to simulated drought conditions. During the experiment, temperature was monitored but not controlled, while relative humidity was not monitored, potentially introducing environmental variation that may have influenced disease development. Furthermore, the environmental conditions and physiological characteristics of young potted trees differ from those experienced by mature trees in the field. Consequently, disease development and the susceptibility of *A. heterophylla* to *N. luteum* infection under natural conditions may differ from that observed in this study. Future pathogenicity assays conducted in climate chambers under controlled temperature and relative humidity, and over longer experimental periods, would provide a more robust assessment of disease development and host-pathogen interactions.

Drought-stressed trees developed smaller lesions than regularly irrigated trees, raising the possibility that reintroducing irrigation following ‘drought’ may enhance pathogen development by facilitating colonization of xylem tissue (Obrador-Sánchez & Hernandez-Martínez, 2020); further studies are required to test this hypothesis. In addition, investigating the effects of other abiotic stressors, such as soil compaction and nutrient deficiency, would improve understanding of the environmental factors influencing the pathogenicity of *N. luteum*.

Finally, *Trichoderma* species were naturally associated with *A. heterophylla*, with two species isolated from six trees in this study. As many *Trichoderma* species are recognized as biological control agents, their presence suggests a potential role in suppressing *N. luteum*. However, the duration of the project did not allow the investigation of their colonization dynamics and antagonistic activity, representing a promising direction for future research.

## Conclusion

Among the fungal species isolated from *A. heterophylla*, *N. luteum* was selected for further pathogenicity testing. All trees inoculated with this fungus developed lesions, with more extensive lesion size observed in watered trees compared with trees experiencing drought. While *N. luteum* appears to contribute to *A. heterophylla* decline, the results suggest that it is unlikely to be the primary causal agent. Further pathogenicity tests, including longer-term experiments and co-infection by other fungi – notably *Nigrospora* species and *A. alternata* – are necessary to determine the full role of pathogenic fungi in tree decline. Correct identification of pathogens can help in the development of potential treatments and management strategies. Regular monitoring and proactive management of *A. heterophylla* is recommended, along with further investigations into fungal communities in the upper canopy.

## Data Availability

No datasets were generated or analysed during the current study.
